# Supplementary material for: Are carbon emissions trading and green financial instruments synergistic? -Comprehensive quantitative research based on content analysis
Source: PLoS One. 2024 Mar 7;19(3):e0298601. doi: 10.1371/journal.pone.0298601 (PMC10919601; doi:10.1371/journal.pone.0298601)
Supplement: S1 Data — (ZIP) [file pone.0298601.s001.zip › Data/地级市Z_F工作报告.zip!/║┌┴·╜¡/║┌║╙╩╨/└┤╫╘╣1⁄2╓┌║┼í╛┬φ┐╦╩2╛▌═°í┐_2003.docx]

各位代表:

现在，我代表市人民政府，向大会报告工作，请予审议，并请市政协委员和其他列席会议人员提出意见。

一、过去五年和2002年工作回顾

市第二届人民代表大会第一次会议以来，全市人民在省委、省政府和市委的正确领导下，认真贯彻党的基本路线、方针和政策，按照“二次创业、兴边富民强市”的要求，克服诸多困难和不利因素，实现了经济建设和社会各项事业稳步发展。预计2002年国内生产总值比1997年增加12.9亿元，年递增5.3%；累计完成社会固定资产投资96.8亿元，实现社会消费品零售总额127.1亿元，年递增7.1%和5.3%。经济结构得到优化，三次产业比例由1997年的45.9:21.2:32.9调整到2002年的32.8:20.9:46.3。一批重点项目相继开工建设并投入使用，202国道黑河至北安段改造工程实现预期目标，北黑地方铁路扩能改造和黑河机场4C级改造基本完成，山口水利枢纽工程和宝山、象山电站建成并投入运营，北五公路、卧牛湖公路、碾北公路交付使用，黑河、逊克、嫩江堤防工程和黑河市区大水源、五大连池引双入青工程相继竣工。对外开放不断扩大，累计实现外贸进出口总额89125万美元，年递增3.7%；完成对俄合作和外商投资项目73项3500万美元；落实招商引资项目1865项，到位资金29.3亿元。各项改革进一步深化，国有企业改制面达到90%，农村二轮土地承包、撤乡并村和农村税费改革顺利完成。城乡建设步伐加快，电网、道路、供排水等基础设施建设及绿化、美化、亮化工程顺利推进，一批标志性小区和公园陆续建成。城镇和农村人均住房面积比1997年分别增长15.6%和12%，农村住房砖瓦化率达到59.9%。城市化水平达到35.3%。教育、科学、文化、卫生、体育等各项社会事业取得新的进步。6个县（市）区全部通过国家“普九”验收，适龄儿童入学率达到99.8%。5年来人口自然增长率下降3个千分点，生育符合政策率提高3个百分点。广播、电视覆盖率分别达到94.3%和91.4%，农村实现了村村通电话。双拥工作取得新成果，进入省级“双拥模范城”行列。

2002年，全市上下牢牢把握发展主题，解放思想，抢抓机遇，迎难而上，国民经济和各项社会事业得到了进一步发展。

国民经济实现较快速度增长。国内生产总值预计实现89.6亿元，比上年增长8.5%，创五年新高。农业增加值实现16亿元，增长9.8%。规模以上工业增加值实现近5.1亿元，增长13.5%，首次实现两位数增长。第三产业增加值实现41.5亿元，增长9.4%。财政一般预算收入完成3.7亿元，按可比口径增长18.3%。社会消费品零售总额达28.8亿元，增长8%。

重点项目建设成效显著。全市确定的77项（其中市区确定11项）重点项目建设全面启动。列入省财源建设项目51项，已启动48项。黑河热电厂扩建完成主体工程，黑河机场路、绥北路开工，富地营子水库完成大坝截流，乌一电站实现并网发电，对俄购电项目和新兴基础原材料加工区建设前期工作稳步推进，北安完达山乳业、九三油脂、爱辉铃田马铃薯全粉、嫩江大豆蛋白纤维等一批产业化龙头项目相继建设或投入生产，黑龙江公园续建工程竣工，瑷珲历史陈列馆建成开展。

对外开放取得新进展。对外贸易按照“五个延伸”要求，积极应对国内外市场和政策变化，实现进出口总额14419万美元，增长12.8%。改善口岸环境有较大突破，口岸收费项目减少，收费额大幅度下降。我市还被国家确定为全国两个实施外向型农业“走出去”战略试点城市之一。接待国内外旅游者67.1万人次，增长6.1%。签订招商引资合同135项，落实120项，到位资金4.31亿元，其中超千万元项目13项。

城乡建设迈出新的步伐。全市基本建设新开工面积68万平方米，增长13.3%。新建给排水管线12460延长米，铺装人行道板12.6万平方米，新增绿地面积22万平方米。市区绿化覆盖率达到11%，人均绿地4.3平方米。城市和农村人均住宅面积分别达到9.02平方米和18.06平方米，农村住房砖瓦化率比上年提高1.14个百分点，自来水普及率达到75%。

各项社会事业稳步发展。改造中小学危房16857平方米，新建校舍26881平方米；师专艺术楼和综合楼、黑河中学综合楼、黑河第五小学、教育大厦相继建成并投入使用。52个涉及高新技术、现代农业、国际科技合作的项目已全部启动实施。文化事业和文化产业有新的发展，群众性文化生活更加丰富多彩。卫生改革不断深化，卫生基础设施逐步完善，市中心血站、市第一医院住院部大楼主体工程相继完工。其他社会事业也都有较大进展。

人民生活水平进一步提高。城镇居民人均可支配收入达到4781元，农民人均纯收入达到2393元，分别增长16.6%和7.5%。城乡居民储蓄余额达到97亿元，比年初增长24%。城镇登记失业率为4%，下岗职工再就业率达到44%，养老、失业保险参保率和城市居民最低生活保障率分别达到100%、99%和100%，在岗职工工资、下岗职工基本生活保障金、城镇居民最低生活保障金和企业离退休职工基本养老金发放率均达到100%。

政府自身建设不断加强。自觉接受市委领导，认真贯彻落实市委重大决策，主动接受人大法律监督、政协民主监督，坚持依法行政，努力推进各项工作。进一步转变政府职能，行政审批事项由550项减少到225项，减少57.5%。扩大了“一条龙”办公、“一站式”服务范围，“公示制”、“承诺制”得到落实。切实转变工作作风，减少会议、文件和不必要应酬，通过深入基层调查研究、现场办公解决实际问题，提高了工作效率。加大了督办检查力度，对重点工作落实情况进行定期通报和反馈。全年共督办事项720项，是上年的2.6倍。整顿市场经济秩序取得阶段性成果。加强了廉政建设，认真纠正了部门和行业不正之风。

各位代表，过去的五年，是全市人民解放思想，大胆实践，努力探索符合黑河市情发展道路的五年；是适应形势变化，抢抓机遇，积极推进改革开放的五年；是经受严峻考验，战胜各种自然灾害，经济社会发展取得新成就的五年。这些成绩的取得，是全市各族人民在市委正确领导下团结奋斗、辛勤工作的结果；是各级人大、政协监督支持和各民主党派、工商联、无党派人士及各人民团体真诚合作的结果；是驻军部队、武警官兵、公安干警强有力保障和中省直各单位共同努力的结果。在此，我代表市人民政府，向为黑河发展做出贡献的领导和同志，向所有关心支持黑河事业的各界朋友表示衷心感谢，并致以崇高敬意！

过去五年，全市经济社会各项事业虽然取得了长足发展，但还存在不少困难和问题。主要是：思想解放不够，抢抓机遇和开拓创新意识不强；经济发展环境不优，阻碍经济发展的各类问题还比较突出；经济总量小，发展速度不快，旅贸牵动的作用还不强；财源基础薄弱，收支矛盾突出；民营经济不活，在国民经济中所占比重偏低；城乡低收入人群增多，就业压力较大；在一些部门和工作人员中仍然存在官僚主义、形式主义和消极腐败现象，等等。对此我们将高度重视，并逐步加以解决。

二、今后五年工作目标和2003年工作任务

全面建设小康社会的宏伟目标，为黑河发展指明了方向。我市属欠发达地区，人均GDP远远低于全省、全国平均水平，同步实现小康目标的压力更大，任务更艰巨。未来五年，我们必须增强忧患意识和赶超意识，把握发展机遇，积极应对挑战，以党的十六大精神为指针，高举邓小平理论伟大旗帜，全面贯彻“三个代表”重要思想，坚持“发展为主，效益第一”原则，深入实施旅贸牵动和可持续发展战略，千方百计发育新产业、增创新优势，加快产业化、工业化、民营化和城镇化步伐，促进经济社会协调发展，不断提高人民群众生活水平，为全面建设小康社会积攒发展后劲，奠定坚实基础。今后五年主要预期目标：国内生产总值年递增9%，一、二、三产业分别年递增3.5%、11%、12%；财政一般预算收入年递增10%左右；全社会固定资产投资年递增11%；城镇居民人均可支配收入和农民人均纯收入年递增12%和 5%；城镇登记失业率控制在5%以内，下岗职工再就业率达到50%以上。为实现上述目标，在工作中必须重点把握以下几个方面：

优化产业结构。第一产业打特色牌，大力发展绿色农业、畜牧业和林业经济；第二产业走优势路，积极发展资源精深加工和出口导向型工业，培育一批具有较强竞争力的骨干企业和著名品牌；第三产业上快车道，加快发展旅游业和现代服务业。加强与区域内农垦、森工合作，合理配置生产要素，坚持优势对接，龙头共用，资源共享，推进区域经济一体化协调发展。

实施项目牵动。围绕经济抓财源，围绕财源抓项目，建成一批事关发展全局和人民生活的重点项目，扩大经济总量，提高经济效益，增加财政收入，积蓄经济发展实力和后劲。

培育发展主体。深化国有企业产权制度改革，建立现代企业制度，提高企业素质，增强企业活力。扶强扶壮民营经济，推动其快速发展并成为主体力量。引进投资和经营主体，壮大整体经济实力。

扩大对外开放。贯彻落实省委对外开放“五个延伸”的要求，坚持“走出去”，以经济技术合作为切入点，做大做强做实一批项目，牵动对俄经贸合作向更深层次、更宽领域发展。坚持“引进来”，吸引国内外人流物流资本流向黑河聚集，形成全方位的对外开放格局。

营造良好环境。牢固树立抓环境就是抓发展的思想，对经济发展环境进行全面整治。综合运用行政、经济、法律手段，建设良好的政策、法制、服务、信用等软环境。加大投入力度，不断改善城市、口岸、交通等硬环境，从根本上解决制约发展的“瓶颈”问题。

坚持协调发展。深入实施科教兴市和可持续发展战略，控制人口增长，保护生态环境，促进经济与人口、资源、环境协调发展。推进民主法制和精神文明建设，大力发展社会各项事业，努力实现“三个文明”共同进步。

各位代表，2003年是深入贯彻十六大精神、启动全面建设小康社会伟大工程的第一年，也是本届政府开局之年。我们要按照市委二届十一次全委（扩大）会议的总体要求，统筹兼顾，突出重点，攻坚克难，真抓实干，努力推动全市经济和社会快速发展。预期目标：国内生产总值增长8%，其中农业增加值增长3.5%，规模以上工业增加值增长6%，外贸进出口总值增长10%；财政一般预算收入增长5%以上；固定资产投资增长10.5%；社会消费品零售额增长8%；农民人均收入增长4.5%。

（一）坚持产业化方向，构筑市场农业发展格局。以市场为导向，打绿色牌，走特色路，努力提高农产品的市场竞争力，增加农民收入。加快结构调整步伐。结合实施“大豆振兴计划”，扩大“双高”大豆和专用大豆种植面积，继续推进高油大豆、马铃薯、饲料牧草、经济作物、柞桑蚕五个百万亩基地建设，种植面积达到360万亩。大力发展绿色食品产业，绿色食品种植面积达到150万亩。把发展牛羊经济作为结构调整的重点，实施“奶业振兴计划”，逐步扩大奶牛、黄牛、绒山羊等生产规模，使畜牧业产值占农业总产值比重达到20%以上。全面实施退耕还林工程、森林生态效益补助试点工程、野生动植物保护和自然保护区建设工程、速生丰产用材林基地建设工程，抓好重点火险区综合治理，加快发展林业经济。积极推进产业化经营。做大做强完达山乳业、铃田马铃薯、九三油脂、嫩江鑫珠、五大连池米业等龙头企业，创造条件启动华辉等加工企业，提高各类加工企业对生产基地的带动能力。积极发育农畜产品市场，发展“订单农业”，提高农畜产品与市场的对接度。引导、支持各类经济合作组织和经纪人队伍发展，提高农民进入市场的组织化程度。改善农业生产条件。深入实施良种化工程，推进专用品种生产基地建设。加强农业标准化监测，实现大豆、小麦、玉米、马铃薯等主栽作物品种标准化种植。积极推广农业先进实用技术，加快农机具更新改造步伐，提高农民素质和科学种田水平。加强农业信息网建设，拓宽技术推广、信息传递和农产品销售渠道。加快农业综合开发，抓好水利、道路建设，实行山水林田路和小流域水土流失综合治理，完成水土保持治理面积8.6万亩。大力发展节水农业，搞好节水灌溉示范项目，全市节水灌溉面积达到10万亩。加强耕地保护。认真落实党的农村政策。深化粮食流通体制改革，积极培育粮食市场。依法推进和完善土地流转机制，促进土地规模经营。巩固税费改革成果，进一步减轻农民负担，保护农民利益。

（二）加快改革与发展步伐，壮大地方工业经济。树立工业立市思想，全力推动工业经济总量上规模、质量上档次、发展上水平。做大做强支柱产业。确保对俄购电项目如期实施，加快新兴基础原材料加工区建设，争取7月份开工生产。积极推进与新加坡亿达航务有限公司合作，加快五大连池矿泉水开发和市场开拓。围绕粮油和畜产品搞好深加工。利用丰富的北药资源，加快推进产品开发，使北药资源优势尽快向经济优势转化。启动金水30万吨煤矿及一批矿井建设，加快西部煤田开发。招商引资争取关鸟河水泥厂早日开工。推进西沟抽水蓄能电站、乌二、乌三水电站、逊克富强和黑河三道湾子岩金矿等项目前期工作。搞好国有资本运营。积极推动逊克4个水电站包装上市，抓好西沟水电公司股份出售。加快东方红、黑宝山、西岗子煤矿和多宝山铜矿等12户国有工业企业改制，尽快完成国有资本退出。对已改制企业，要通过股权出售、民有民营等方式，转换内部机制。抓好企业技术改造与产品创新。用高新技术改造和提升传统产业，重点抓好热电厂、三精药业等22个技术改造项目和生物调解剂、小型大豆联合收割机等5个技术创新项目的组织实施。培育和发展知名品牌，提高产品市场占有率。加强企业信息化建设。注重信息化在企业经营管理中的应用，带动企业整体素质和竞争力的提高，以信息化带动工业化。重点抓好黑河热电厂、黑宝山煤矿、西沟水电公司等企业信息化建设。强化工业经济调控和服务。对规模以上企业进行重点调控，把握全市工业经济运行动态，及时解决各种困难和问题。集中力量抓好重点行业和重点企业扭亏增盈，提高经济运行质量。

（三）深入实施“五个延伸”，推动对俄经贸合作战略升级。把对俄经贸作为重要牵动力量，外资外经外贸相互渗透，一二三产业共同参与，举全市之力整体推进。以项目合作带动经贸发展。组织各县（市）区、辖区内各农场局、林业局充分利用黑河口岸优势积极开展对俄经贸合作。精心抓好对俄购电项目组织实施，带动地方工业产品出口。积极开展森林采伐和木材加工合作，争取年内采伐木材30万立方米，加工木材10万立方米，带动木材加工和进口，促进锯材出口和转口。合作经营中俄果蔬批发市场，增加设施和品种，完善配送功能，带动水果蔬菜出口。抓好爱辉、逊克外向型农业合作园区建设，开展在俄土地承包承租、畜牧养殖和粮油加工，带动农畜产品进出口。树立大口岸意识，充分发挥联检部门职能作用，为国内外企业开拓俄罗斯市场提供服务。加强市场分析研究，寻求国内外合作伙伴，探索合作项目。拓展利用外资领域，提高利用外资规模和质量。多种贸易形式并举。在抓好钢轨、木材、服装、轻工产品等传统商品进出口的同时，增加价值和科技含量高商品的进出口，扩大边境贸易规模。放大互贸区功能并向市区延伸，引进新的经营主体搞活国贸城经营，推进民贸公司化运作。增加甜菜粕、豆秸粕、速冻土豆、木制品等地方产品出口，发展一般贸易。引进和扶强经贸主体。进一步放开外贸企业经营权，吸引国内有实力的民营企业参与对俄经贸合作，力争引进3—5家年贸易额超千万美元的企业到黑河注册经营。对年过货额300万美元以上中俄边贸企业和业绩较好的市属骨干企业进行重点扶持，增强外经贸主体活力。在现有基地和境外市场的基础上，组织企业到俄腹地与更多有实力的俄罗斯零售商、生产商、代理商建立稳定的合作关系。优化口岸环境。加强两岸政府间协调，共同解决经贸合作中的问题。认真抓好两岸政府会谈纪要的组织实施，专题推进具体项目，共同把议定事项落到实处。推行界江车船运输市场化运营，推动中省直部门和俄阿州同步降费。进一步降低地方铁路运价，增加外运车皮，解决运输瓶颈制约。完善两岸保险理赔、经济仲裁措施，做好黑河与阿州本币结算业务试点工作。保护在俄中国公民的合法权益。积极做好黑龙江大桥建设前期工作。加快202国道建设，力争年底全线通车。进一步完善口岸功能，争取尽快开通黑河国际空港。

（四）突出发展旅游业，带动第三产业提档升级。精心组织实施旅游发展总体规划，以景区景点建设带动线路开发和市场开拓。发展壮大旅游主体。面对92版旅游护照到期、中俄互为旅游目的地国等新形势，引进培育具备经营资格和经济实力的经营主体，建立适应市场竞争需要的管理体制和运行机制，不断扩大边境旅游规模。加快景区景点建设。抓住国债资金支持旅游基础设施建设的机遇，做好景区景点项目编制和争取工作。市区重点抓好瑷珲历史陈列馆功能完善，筹划和启动北方生态观光园、卧牛湖滑雪场建设。加快推进五大连池风景名胜区自然保护区的保护、开发和建设，申报世界自然遗产，争创4A级风景区。积极协调俄阿州、布市进一步加强景区景点建设，增强边境游的吸引力。将国内游与边境游有效对接，延长线路，丰富内容，增加游客在黑河的旅游时间。加强旅游品牌培育和旅游产品营销。打造具有黑河特色的旅游品牌，加大宣传促销力度，精心组织好旅游文化节、饮水节、冰雪节及各种会展，开发假日旅游和会展购物旅游，拓展国内外旅游市场。加快开发玛瑙制品、桦树皮制品等特色旅游纪念品。预期接待国内外游客75万人次，增长8.6%；边境旅游达到10万人次，增长25%。大力发展服务业。运用现代经营方式和服务技术改造提升商贸、交通、餐饮等传统服务业，提高服务质量和水平。积极发展社区服务业，健全服务网点。加速发展信息、金融、保险、会计、法律等现代服务业和便民超市、连锁经营、物流配送、电子商务等现代物流业。发挥城市中心市场的辐射和带动作用，以市场建设为突破口，促进服务行业结构优化，带动服务业发展。通过努力，使第三产业增加值实现46亿元，增长11%。

（五）拓宽领域和空间，加速民营经济发展。从改善环境、强化服务入手，促进民营经济大提高、大发展。预期民营经济增加值、税金分别增长10%以上。确立主体地位。真正把民营经济抓在手上，作为主体力量加快发展。贯彻落实国家、省有关吸引和扩大民间投资政策，最大限度地激活民间资本，千方百计把民间资本转化为创业资本。制定鼓励党政机关干部、事业单位人员领办、创办民营企业的优惠政策。加大扶持力度。坚持普遍支持和重点扶持相结合，每个县（市）区都要按照投资额和纳税额选择确定一批重点企业和大户进行重点扶持，实行特惠制。降低民营企业准入门槛，除国家法律、法规明令禁止的行业外，所有行业全面放开。放宽注册资本条件，允许新设立的民营公司注册资本分期到位，并在经营场所、冠名条件等方面给予适当放宽。金融部门要增大对民营经济信贷投放规模，在贷款上给予支持。充分发挥中小企业担保中心作用，与金融部门联手加大资金投放，解决好中小企业贷款难问题。打破所有制界限，帮助民营企业向上争取项目和资金。建立民营经济服务中心，帮助民营企业解决生产经营过程中的实际困难，为民营经济提供有效服务。实行“收费明白卡”制度，制止“三乱”，维护企业和业户的合法权益。提高企业素质。鼓励民营企业向生产型、科技型、外向型发展，引导民营企业参与国有企业改革、绿色食品开发、外向型经济、城市建设、现代服务业，不断扩大发展规模。加快民营企业机制转换和技术创新步伐，做大做强一批具有较强牵动作用的民营企业群体。引导和监督企业依法经营，照章纳税，促进企业健康发展。

（六）创造优良经济发展环境，促进招商引资实现突破。坚持财政收入保运转、向上争取搞建设、招商引资求发展，进一步改善环境，强力推进招商引资工作。实施全民招商。把招商引资作为加快发展的牵动力量高度重视，强化组织领导，实行一把手工程，并将招商任务指标量化，层层落实责任，认真兑现奖惩。坚持多元驱动，在强化各级领导和部门招商主导的同时，注重把招商主体向基层企业和群众延伸，动员和组织全市范围各行各业开展大招商。突出招商重点。围绕资源开发、龙头企业建设和引进民营经济大户、外经贸主体，选择重点地区、重点行业、重点企业进行定向定位招商。立足资源和区位优势，以项目为依托，强化项目库建设。切实做好招商项目前期准备工作，落实必要的前期专项资金。注重会展招商，精心组织参加哈洽会，办好大黑河岛经贸洽谈会和俄阿州中国名优产品展销会等大型招商活动。改善招商环境。以营造良好的经济社会发展环境带动招商引资深入开展。推行首问责任制、服务承诺制、限时办结制、失职追究制、否定报备制和无偿代办制，简化办事程序和环节，提高服务效率和质量。加快政务超市建设，建立便民大厅，强化一站式办公，一条龙服务。抓好信用环境建设，着力培养信用政府、信用企业和信用个人，规范市场经济秩序，加大对失信行为的依法惩处力度。增强执行政策的灵活性和创造性，把各项招商政策落实到位。对人为设障和各类损害外来投资者合法权益的从严惩处。

（七）坚持建设与管理并重，进一步提高城市化水平。把城市作为最大国有资产来经营，不断完善城乡基础设施，加快城乡建设步伐。科学制定并严格执行规划。强化城市规划职能，组织编制城市近期规划及通江路、王肃街等重点区域的详控规划，严格按规范标准编制部分小区详控规划。抓紧完成小城镇规划编制工作。坚持依法办事，维护规划的严肃性和权威性，坚决杜绝私批乱建、见地插楼问题。活化城市资产。成立黑河市土地储备整理中心，对城市土地进行收储、整理和销售，实现市场化运作。改革市政基础设施和公益设施有偿转让办法，对冠名权、广告权等无形资产实行商业化运营，实现有形化运作。加快基础设施建设。通过向上争取和招商引资增加城市建设投入。引导城市开发与市政建设、公益设施建设相结合，吸引更多的民间资金投入城市建设。抓好沾五公路等7条县乡公路建设，不断完善城乡路网。抓住国债资金向城市建设倾斜的机遇，争取黑河污水处理厂尽快开工建设，推动嫩江、五大连池、孙吴、逊克等地供热、给排水项目的组织实施。抓好市区重点区域的绿化、亮化、美化、洁化工程。继续开展公园及街路绿地认领、认建工作。促进房地产业稳步发展。加速新区开发，同时兼顾老城区改造，全市新开工建筑面积46万平方米，其中市区20万平方米。面向中低收入居民积极开发经济适用住房。科学制定房屋动迁补偿标准，活跃房地产开发市场。搞活住房二三级市场，培育住房消费热点。实施“阳光工程”，进一步整顿和规范建筑市场。切实加强城市管理。对城市管理行政执法权实行相对集中，建立高效、顺畅城管体制。继续推行“门前包保”责任制，对城乡结合部、城市出口等地段进行专项治理。抓紧完成物业服务区划，启动现有住宅小区区域性改造，扩大覆盖范围，增加服务内容。加快生态市建设步伐。以治烟、治污水、治垃圾为重点，对城市环境进行综合整治。加强对工业污染管理，从严控制污染物排放。推广使用清洁燃料，减少大气污染。严厉查处非法采金，防止植被破坏、河流污染和水土流失。抓好自然保护区建设。稳步推进小城镇建设。以现有国家、省级试点小城镇为重点，全面加快基础设施和住宅建设，逐步完善道路、给排水、电、热等设施。推进城镇户籍制度改革，转移农村剩余劳动力，促进人口向城镇有序流动。加快小城镇产业发展，以工业化带动城镇化。

（八）做好财政金融工作，支持经济社会发展。面对增支因素多、运转难度大的实际，进一步树立过紧日子思想，广辟渠道，开源节流，确保收支平衡。强化税收征管。建立经营业户数据管理、税源登记、税费征缴网络管理中心，在突出抓好骨干税源征管同时，切实加强零散税种征收。继续开展定额复查和漏征漏管户清理，严格依法治税，集中整治偷逃欠骗抗税和漏征漏管、越权减免等问题。挖掘潜力，做到应收尽收。认真执行收支脱钩和收缴分离制度，强化监管稽查，确保非税收入比上年增长10%。优化支出结构。按照公共财政要求，严格支出顺序，集中财力确保职工工资、离退休人员基本养老金和医疗费、下岗职工基本生活费、城市居民最低生活保障金及时足额发放。大力压缩一般性支出，保障党政机关正常运转，并尽可能为经济社会发展提供财力支持。加快财源建设。下大力气抓好市县乡三级财源建设，对已纳入省重点财源扶持范围的项目全力推进，如期达产达效。抓紧谋划论证一批新项目，使纳入省财源建设盘子的项目比上年有进一步增加。深化财政体制改革。全面推行国库集中收付制度，不断扩大政府采购范围，加快推进部门预算编制改革，深化“收支两条线”管理改革，强化预算约束。逐步深化国有资产管理体制改革，经营和管理好国有资产，推进国有资产市场化运作进程。加大对财政重点支出项目和专项资金使用的监督检查和审计。加大向上争取力度。认真研究和把握国家、省产业政策和投资方向，抓住各种有利机遇，动员和组织方方面面力量，千方百计做好向上争取资金工作，弥补财力和事业发展资金缺口。

做好金融工作。金融部门要加大对经济发展支持力度，增加对农业、重点项目、对俄经贸、民营经济等重点领域的信贷投放规模。创新信贷服务品种，引导和刺激各种新兴消费。积极做好核呆工作，降低不良资产比例。地方政府要大力支持金融业发展，加强银地合作，打击逃废债行为，维护金融安全。

（九）坚持协调发展，实现社会各项事业全面进步。统筹安排，把物质、政治和精神“三个文明”建设作为统一目标同步推进。加强思想道德和法制建设。按照《公民道德建设实施纲要》要求，加强公民道德教育和爱国主义教育，提升社会整体文明水平。深入开展“四五”普法，加快依法治市步伐。加强基层政权建设，扩大基层民主。优先发展教育。遵循适度超前发展原则，努力增加教育投入，保障教育经费稳定增长。深化教育改革，合理配置教育资源，优化教育结构，提高教学质量和水平。巩固和提高“普九”成果，加大 “控辍”力度。完成18所中小学校舍改造，改善办学条件。积极开展职业技术教育和成人教育，培养实用技术型人才。大力发展高中教育和高等教育，推进信息技术教育。深入实施“科技兴市”战略。注重人才的引进、培养和储备。加大科技投入，强化科技创新对全市经济社会发展的支撑。精心组织绿色食品、矿产和农副产品精深加工、环保等领域的技术开发和应用，抓好省级以上重点科技计划项目的组织实施。推进合作区中俄科技园区争取建设工作。做好卫生和计划生育工作。深化卫生体制改革，全面实行药品集中招标采购。强化农村卫生工作，推进农村乡镇卫生管理体制改革，改善农村卫生医疗条件，实行大病统筹合作医疗制度。加强卫生基础设施建设，完善社区卫生服务网络。稳定低生育水平，全市人口出生率控制在9.2‰以内，人口自然增长率控制在4.2‰以内，生育符合政策率达到95%以上。做好民族宗教工作。大力发展少数民族经济，扶持人口较少民族发展。精心组织好鄂伦春族下山定居50周年迎庆活动。依法加强宗教事务管理，坚决取缔非法宗教活动。大力发展其他各项社会事业。加强文化市场管理，净化文化环境。广泛开展群众体育活动，努力提高优势项目竞技水平。深入开展“双拥共建”活动，争创国家级“双拥模范城”。重视发展老龄事业，支持残疾人事业发展。深入实施妇女儿童发展规划，保护未成年人合法权益。进一步做好广播电视、气象、地震、地方志、档案、文博图等项工作。

（十）采取综合配套措施，确保社会安全与稳定。妥善处理改革、发展与稳定的关系，营造安全稳定社会环境。促进下岗失业人员再就业。引导下岗失业人员转变观念，自谋职业，灵活择业。大力发展以增加就业岗位为主的中、小企业，通过发展经济增加就业岗位。挖掘企业内部安置潜力，积极开辟就业领域。完善就业服务体系建设，开展多层次再就业培训，健全劳动力市场信息网络，为下岗职工再就业提供有效服务。认真落实国家、省促进再就业优惠政策，抓好地方配套政策制定、完善和落实。进一步做好社会保障工作。完善养老、失业、医疗、工伤、生育等社会保险保障体系，扩大覆盖范围，提高社会化管理水平，建立稳定、有效的社会保障基金筹措和管理机制。抓好城市低保工作，实现城市低保对象应保尽保。大力扶助社会弱势群体，深入开展“走进千万家”、城镇“一帮一”和为农村贫困户送温暖活动，努力为弱势群体排忧解难。深入开展社会治安综合整治。继续开展打黑除恶专项斗争，加大案件侦破力度。加强制度建设，做好矛盾纠纷排查工作，预防职务犯罪和青少年犯罪。依法坚决打击“法轮功”组织和各种敌对势力不法活动。全面落实安全生产责任制，加大整治力度，坚决消除隐患，避免各类安全事故发生。妥善处理群众来信来访。坚持“分级负责、归口管理”原则，完善各级政府领导电话、信件和来访接待日制度，及时化解矛盾，解决实际问题。落实领导包案和“一案三查”制度，逐一落实“包稳控”措施，减少集体上访和越级上访。加强国防建设。普及国防教育，提高国防意识。强化边防、人防、民兵预备役建设。加大边境管理力度，维护边境秩序，确保边境安全与稳定。

各位代表，今年在抓好上述工作的同时，在市区范围内，要集中精力抓好事关人民群众切身利益的7件实事：一是推动黑河师专专升本，创建黑河学院，力争年内通过国家高评委验收；二是推进黑龙江公园续建工程，完成原王肃公园改造；三是兴建黑河体育馆；四是完成黑河市第一医院住院部大楼工程；五是完成中央街东段、环城路沥青罩面工程；六是改造火车站站前广场；七是完成热电厂改造工程，取消部分小区供热。各县（市）区也都要根据各自情况，尽心尽力为人民群众办一些实事好事。

三、切实加强政府自身建设，不断提高依法行政水平

要完成今后五年任务，政府必须全面加强自身建设，做到行为规范、运转协调、公正透明、廉洁高效。努力建设学习型政府。深刻领会邓小平理论和“三个代表”重要思想的精髓，准确把握国情和市情，提高科学判断形势的能力。认真研究市场经济规律，找准国家政策与经济欠发达边境地区的结合点，创造性开展工作，抓住机遇加快发展，提高驾驭市场经济的能力。努力学习世贸组织规则和国际贸易、公共管理和现代科技等知识，提高政府整体素质。努力建设法制型政府。自觉接受市委正确领导，认真贯彻市委决策。主动接受人大法律监督和政协民主监督，认真办理人大代表议案、建议、批评、意见和政协委员提案。加强法制教育，增强公务员特别是各级领导干部的法律意识，提高执法人员素质。推行行政执法责任制，规范执法，严格执法，文明执法，切实做到依法行政。努力建设服务型政府。进一步转变职能，继续减少行政审批事项，减少政府对微观经济的直接管理和资源的直接配置。侧重于解决各种梗阻问题，为经济发展服务；侧重于政策引导和环境建设，为市场主体服务；侧重于为百姓办实事、解难题，为人民群众服务。积极推进政务信息化建设，实现政府与企业、社会、公众信息资源共享。努力建设务实型政府。转变观念，增强抓落实的主动性；结合实际，增强抓落实的创造性；求真务实，增强抓落实的实效性。完善工作推进机制，强化制度保证。健全目标责任管理体制，把各项任务层层分解、层层定标，细化量化到每个部门，并明确具体责任人和完成时限。强化督促检查制度，跟踪问效，定期通报和反馈。建立健全奖惩制度，把工作落实情况作为考核政绩的突出内容，以绩取人。转变作风，深入基层开展调查研究、现场办公，解决实际问题。注重提高效率，减少环节，精简会议和文件。努力建设廉洁型政府。务必保持谦虚、谨慎、不骄、不躁的作风，务必保持艰苦奋斗的作风，提倡勤俭办事业，反对奢侈浪费。健全行政监察、审计监督等管理和监督机制，坚决查处和严厉打击各种以权谋私和贪污腐败行为。下大力气纠正行业不正之风，促进政风和社会风气好转。

各位代表，未来五年是我市全面建设小康社会的重要发展机遇期。让我们解放思想，与时俱进，团结奋斗，真抓实干，全力开创黑河经济社会各项事业发展新局面！
